# Swedish rape offenders — a latent class analysis

**DOI:** 10.1080/20961790.2020.1868681

**Published:** 2021-02-22

**Authors:** Ardavan Khoshnood, Henrik Ohlsson, Jan Sundquist, Kristina Sundquist

**Affiliations:** Department of Clinical Sciences, Center for Primary Health Care Research, Lund University, Malmö, Sweden

**Keywords:** Forensic sciences, Sweden, crime, sex crimes, rape, offender characteristics, crime prevention, latent class analysis

## Abstract

Sweden has witnessed an increase in the rates of sexual crimes including rape. Knowledge of who the offenders of these crimes are is therefore of importance for prevention. We aimed to study characteristics of individuals convicted of rape, aggravated rape, attempted rape or attempted aggravated rape (abbreviated rape+), against a woman ≥18 years of age, in Sweden. By using information from the Swedish Crime Register, offenders between 15 and 60 years old convicted of rape+ between 2000 and 2015 were included. Information on substance use disorders, previous criminality and psychiatric disorders were retrieved from Swedish population-based registers, and Latent Class Analysis (LCA) was used to identify classes of rape+ offenders. A total of 3 039 offenders were included in the analysis. A majority of them were immigrants (*n* = 1 800; 59.2%) of which a majority (*n* = 1 451; 47.7%) were born outside of Sweden. The LCA identified two classes: Class A — low offending class (LOC), and Class B — high offending class (HOC). While offenders in the LOC had low rates of previous criminality, psychiatric disorders and substance use disorders, those included in the HOC had high rates of previous criminality, psychiatric disorders and substance use disorders. While HOC may be composed by more “traditional” criminals probably known by the police, the LOC may represent individuals not previously known by the police. These two separated classes, as well as our finding in regard to a majority of the offenders being immigrants, warrants further studies that take into account the contextual characteristics among these offenders.

**Key points**
- Rape, aggravated rape, attempted rape or attempted aggravated rape (rape+) are increasing in Sweden.
- The majority of those convicted of rape+ are immigrants.
- LCA identifies two classes of rape+ offenders: LOC and HOC.

## Introduction

Sexual crimes against women, including rape, are universally affecting women all over the globe [1]. Moreover, it is likely that both rape and other types of sexual crimes are highly underreported according to data from the US [2]. The Swedish National Council for Crime Prevention (In Swedish: Brottsförebyggande rådet or Brå for short) has stated that there were 23 200 sexual crimes reported to the police in 2019, of which 8 820 (38.0%) were rapes and aggravated rapes [3]. According to the annual Swedish Crime Survey for 2019, it was estimated that 5.6% of the total population between 16 and 84 years old (*n* = 452 000) had been a victim of sexual crimes. Not surprisingly, more females (9.4%) than males (1.4%) stated that they were victims of sexual crimes. Based on data from the survey, even though the rates of sexual crime victimization in both females and males seem to have increased during the last years, there are indications of a decline since 2017 [4]. Sexual crime rates based on reports to the police indicate that both sexual crimes in general and rape in particular are rising in Sweden. Rape, which is the most serious sexual crime, increased by 45.4% between 2008 (*n* = 5 446) and 2018 (*n* = 7 920) [5]. With the increase of these crimes, the rate of suspects has also increased. In discussing sexual offences in general, there has been an increase of suspects by around 20% since 2007. For rape, the number of suspects has increased with 29.2% between 2007 (*n* = 2 231) and 2016 (*n* = 2 883). Despite these apparent increases, the number of offenders found guilty of sexual offences have been quite stable between 2008 (*n* = 1 055) and 2017 *(n* = 1 162), as the rate of solved rapes by the police has significantly diminished since 2009 (31%); in 2016 the rate was as low as 11% [5].

Sexual crime is an umbrella term consisting of several different crimes with a sexual content (Appendix 1). The most heinous act of sexual crime is rape, which is also the focus of this study. With the apparent increasing rate of rapes, it is of vital importance to have knowledge and gain information on the offenders committing these crimes, as well as which factors that may act protective and detrimental on their criminal behaviour.

The aim of this study is to use Latent Class Analysis (LCA) to identify different classes of offenders, based on a comprehensive set of characteristics. These offenders were convicted of rape, aggravated rape, attempted rape or attempted aggravated rape, together called rape+, against a woman ≥18 years of age. Sexual offenders have been shown to have both common characteristics as well as significant differences. By identifying different offender classes, common individual characteristics can be detected and contribute to a more secure profiling of rape offenders [6] or even connect certain characteristics to a specific rape motive [7]. It can also contribute to the field of crime prevention, and risk analysis may in a more effective manner focus on individuals being at high risk to commit sexually motivated crimes in order to provide support to these individuals. The information can also be used by the police authority after a crime has been committed in order to draw a profile of the likely offender.

## Materials and methods

We analyzed information on individuals from Swedish population-based registers with national coverage. These registers were linked using each individual’s unique identification number replaced by a serial number to preserve confidentiality. The study is covered by ethical approval from the Regional Ethical Review Board in Lund (Dnr: 2012/795). The database for the LCA was created by selecting all individuals convicted of rape+  between 2000 and 2015 (*n* = 3 431) and were between 15 and 60 years old (392 individuals did not meet this criterion). In total, we investigated 3 039 unique individuals. Rape+ were defined as stated in the Swedish Penal Code. Their penal code chapters and sections, as well as the crime codes as defined by the National Crime Council for Crime Prevention, i.e. Brå [8], are as follows:

- Rape and aggravated rape
  Brå codes: 9635, 9636, 9637, 9638, 9639, 9640, 9641, 9642
- Attempted rape and attempted aggravated rape

Brå codes: 9627, 9628, 9629, 9630, 9631, 9632, 9633, 9634

Based on information from the Swedish population-based registers, we also included individual information on white collar crime, property crime, violent crime, drug use disorders, alcohol use disorders and psychiatric disorders. For a definition see Appendix 2. The registration had to occur prior to the registration for rape in order to be included in the database.

One of the studied variables is “country of birth”. Based on this variable, the offenders are deemed to be first- or second-generation immigrants (Swedish born with one parent born in Sweden, Swedish born with no parent born in Sweden, and born outside Sweden) or Swedes (Swedish born with Swedish born parents). In defining who constitutes an immigrant, we have used the definition presented by the Swedish National Council for Crime Prevention: an individual born outside of Sweden or being born in Sweden with at least one parent born in another country [9] represent first- and second-generation immigrants, respectively.

### LCA

LCA is a statistical algorithm used to analyze clusters of observed variables by constructing categorical unobserved or latent segment classes based on weighted analysis and the average probabilities. The latent classes are used to infer variables whose relationships are not directly observed [10]. The LCA was used to identify homogeneous classes of rape+ offenders based on the observed variables previously defined. We therefore entered, into the LCA, six dichotomous variables (yes/no) for each of the following registration types (white collar crime, property crime, violent crime, drug use disorder, alcohol use disorder, psychiatric disorder) and a three-categorical variable defining the number of sexual criminal convictions (1 conviction, 2 convictions, 3 or more convictions). The number of latent classes indicated by the observed variables was determined by comparing model fit statistics between nested models. Improvement in model fit is indicated by smaller values of the log-likelihood, Akaike’s Information Criterion (AIC), entropy and the adjusted Bayesian Information Criterion. The number of classes is influenced by the number of observed variables, so both empirical (improved model fit) and theoretical (model interpretability) aspects were also considered. Individual subjects were then assigned class membership based on the likelihood of their particular response profile. The LCA was performed using PROC LCA in SAS v.9.4 [11,12].

We then included several external validators at the individual level (year of birth, sex, low education, age at first registration with the sexual crime, country of birth, resilience, IQ, school achievement, income, social welfare and neighborhood deprivation) and at the parental level (sexual crime, psychiatric disorder, white collar crime, property crime, violent crime and substance use disorders) to investigate differences across LCA classes. Chi-square analyses were used to compare categorical variables and one-way ANOVA was used for continuous variables. Thereafter, we examined, by logistic regression, the patterns of the external validators when comparing the two LCA classes.

In a further attempt to validate the LCA, we selected from the Swedish Multi-Generation Registry all Swedish-born full-sibling pairs, born between 1950 and 1995. We linked this database to assigned class membership data from the LCA, treating those in other classes as unaffected. Tetrachoric correlations and odds ratios (ORs) were calculated for sibling pairs across LCA classes. As a validation we would assume that the within-class correlation is stronger than the across-class correlation. All statistical analyses were performed using SAS 9.4 [13].

## Results

### Descriptive data

Between the years 2000 and 2015, a total of 3 039 offenders were convicted of rape+ against a woman (Table 1). The majority of the offenders were men (*n* = 3 029; 99.7%) and the mean year of birth was 1976 (SD 12.3). Close to half of the offenders were born outside of Sweden (*n* = 1 451; 47.7%) followed by Swedish born offenders with Swedish born parents (*n* = 1 239; 40.8%). A relatively small part of the cohort was constituted of offenders being born in Sweden with at least one parent being born outside Sweden (*n* = 349; 11.5%). Table 2 shows from which regions the first- and second-generation immigrants and their parents originate from. Among Swedish born offenders with one parent born outside of Sweden (*n* = 172), the foreign-born parent was mostly born in Western Countries (72.7%) followed by Eastern Europe (11.0%). Regarding Swedish born offenders with no parent born in Sweden (*n* = 177), a high proportion of the mothers and fathers were born in Western countries (40.7% and 33.9%) followed by the Middle East/North Africa (19.8% and 24.0%). The largest group of the study population was found among offenders born outside of Sweden (*n* = 1 451); a significant part was from the Middle East/North Africa (34.5%) followed by Africa (19.1%).

**Table 1.** Descriptive data of the offenders (*n* = 3 039).

Item	Descriptive data
Demographics	
** **Year of birth [mean (SD)]	1976 (12.3)
** **Male sex [*n* (%)]	3 029 (99.7)
** **Age at first registration [year, mean (SD)]	32.3 (11.6)
** **Low education [*n* (%)]	1 134 (37.3)
Country of birth	
** **Swedish born with Swedish born parents [*n* (%)]	1 239 (40.8)
** **Swedish born with one parent born in Sweden [*n* (%)]	172 (5.7)
** **Swedish born with no parent born in Sweden [*n* (%)]	177 (5.8)
** **Born outside Sweden [*n* (%)]	1 451 (47.7)
Socioeconomic status	
** **Neighbourhood deprivation [SDI (SD)]	1.3 (2.3)*
** **Income [mean (SD)]	0.0 (1.0)*
** **Social welfare [*n* (%)]	989 (32.5%)
Conviction and suspicion	
** **1 conviction [*n* (%)]	2 834 (93.3)
** **2 convictions [*n* (%)]	181 (6.0)
** **≥3 convictions [*n* (%)]	24 (0.8)
Prior convictions	
** **White collar crime [*n* (%)]	528 (17.4)
** **Property crimes [*n* (%)]	1 119 (36.8)
** **Violent crime [*n* (%)]	1 402 (46.1)
Psychiatric ill-health	
** **Psychiatric disorder [*n* (%)]	482 (15.9)
** **Drug use disorder [*n* (%)]	637 (21.0)
** **Alcohol use disorder [*n* (%)]	588 (19.3)
Others	
** **Resilience (*n* = 964) [mean (SD)]	−0.5 (1.0)*
** **IQ (*n* = 1 076) [mean (SD)]	−0.6 (1.0)*
** **School achievement (*n* = 1 094) [mean (SD)]	−1.0 (1.1)*

SD: standard deviation; SDI: social deprivation index. *This is a standardized variable with mean 0 and SD 1. Please see more details in the Appendix 2.

**Table 2.** Data on the country of birth regarding the included offenders (*n* = 3 039) or their respective parents.

Country of birth	Data
Swedish born with Swedish born parents [*n* (%)]	1 239 (40.8%)
Swedish born with one parent born in Sweden [*n* (%)]	172 (5.7%)
Eastern Europe	11.0%
Western countries	72.7%
Middle East/North Africa	5.8%
Africa (excluding North Africa)	2.9%
Asia (excluding Middle East) and Oceania	6.4%
Latin America and the Caribbean	1.2%
Swedish born with no parent born in Sweden [*n* (%)]	177 (5.8%)
Eastern Europe	10.7%/11.7%*
Western countries	40.7%/33.9%*
Middle East/North Africa	19.8%/24.0%*
Africa (excluding North Africa)	9.6%/10.5%*
Asia (excluding Middle East) and Oceania	11.9%/12.9%*
Latin America and the Caribbean	7.3%/7.0%*
Born outside Sweden [*n* (%)]	1 451 (47.8%)
Eastern Europe	15.0%
Western countries	7.3%
Middle East/North Africa	34.5%
Africa (excluding North Africa)	19.1%
Asia (excluding Middle East) and Oceania	14.4%
Latin America and the Caribbean	9.7%

*The first number is based on biological mother and the second is based on biological father.

A not negligible part of the offenders (*n* = 989; 32.5%) received social welfare and over a third had low education (*n* = 1 134; 37.3%). Interestingly, a significant part of the offenders had a higher educational level than just 9 years of schooling, and most of them did not receive social welfare. Information on resilience, IQ and school achievements were only available for part of the cohort and these variables showed low levels for each of these respectively, i.e. −0.5 (SD 1.0), −0.6 (SD 1.0) and −1.0 (SD 1.1).

For prior convictions, many of the offenders were convicted of violent crimes (*n* = 1 402; 46.1%) followed by property crimes (*n* = 1 119; 36.8%). A relatively small part of the cohort (*n* = 482; 15.9%) had at least one psychiatric disorder, and one-fifth had alcohol use disorders (*n* = 588; 19.3%) and/or drug use disorders (*n* = 637; 21.0%).

Information regarding the offender's parents were only available for a part of the cohort (*n* = 2 071; 68.1%) (Table 3). The most common prior parents’ conviction was property crimes (*n* = 546; 26.4%), followed by violent crimes (*n* = 386; 18.6%). For rape+ crimes (*n* = 10; 0.5%) the corresponding percentage was much lower. Close to a quarter of the offenders had at least one parent with psychiatric disorders (*n* = 508; 24.5%). The corresponding percentages for alcohol use disorder and drug use disorder were 20.0% and 9.4%, respectively.

**Table 3.** Descriptive data of the included offenders’ parents (*n* = 2 071).

Item	Descriptive data
Prior convictions	
** **Rape+ crimes [*n* (%)]	10 (0.5%)
** **White collar crimes [*n* (%)]	335 (16.2%)
** **Property crimes [*n* (%)]	546 (26.4%)
** **Violent crimes [*n* (%)]	386 (18.6%)
Psychiatric ill-health	
** **Psychiatric disorder [*n* (%)]	508 (24.5%)
** **Drug abuse [*n* (%)]	194 (9.4%)
** **Alcohol abuse [*n* (%)]	414 (20.0%)

### LCA

Class enumeration was conducted by using fit indices (Table 4). There was a significant drop in log-likelihood and AIC when going from two classes to three classes. However, the entropy value also decreased, suggesting that the separation of classes got worse. The model with three classes resulted in a class in between what we below call “high offenders’ class” and “low offenders' class”. As the entropy decreased, we therefore chose to use a two-class model, i.e. Class A — low offenders’ class and Class B — high offenders’ class. Furthermore, in the models there are some indications of conditional dependence for some of the manifest variables. This also strengthens the restriction of the number of classes to two, as local independence often leads to additional classes in the final model.

**Table 4.** Fit indices for latent classes.

Number of latent classes	Log-likelihood	AIC	Adjusted BIC	Degrees of freedom	Entropy
2	−9 467.62	466.17	514.48	174	0.78
3	−9 378.12	305.17	379.06	165	0.64
4	−9 323.77	214.46	313.93	156	0.64

AIC: Akaike's Information Criterion; BIC: Bayesian Information Criterion.

Members of Class A had a high rate of class membership probability (71.9%) while Class B had a low rate of class membership probability (28.1%) (Table 5).

**Table 5.** Assignment probabilities by class.

Assignment probabilities	Class A	Class B
Class membership probabilities	71.9%	28.1%
Item response probabilities		
** **White collar crime	4.6%	50.2%
** **Property crime	16.6%	88.5%
** **Violent crime	29.1%	89.8%
** **Drug use disorder	7.2%	56.1%
** **Alcohol use disorder	6.9%	51.2%
** **Psychiatric disorder	7.2%	38.0%
Number of convictions for the studied crimes		
** **1 conviction	95.5%	91.7%
** **2 convictions	5.7%	6.6%
** **≥3 convictions	0.4%	1.7%

Table 6 shows the comparison of covariates across classes. The majority of the offenders (*n* = 2 268; 74.6%) were found in Class A and constitutes of almost exclusively men (99.7%). Mean age for first conviction was 31.2 (SD 11.6) and more than one-third (35.4%) of the offenders had a low education. A substantial amount of the offenders (62.2%) in Class A were first- or second-generation immigrants, defined as an individual born outside Sweden or in Sweden with at least one parent being born abroad. A clear majority of the low offenders were convicted of rape and aggravated rape (79.8%) followed by attempted rape and attempted aggravated rape (20.6%). In regard to socioeconomic variables, the low offenders had a relatively low rate of receiving social welfare (26.0%). The variables resilience (*n* = 962), IQ (*n* = 1 074) and school achievement (*n* = 1 093) were only available for a small part of the offenders and these variables showed low levels. Statistics on offenders’ parents showed that they, in comparison to Class B's offenders' parents, also were low offenders, with, however, a relatively high part of them being diagnosed with some form of psychiatric disorder (21.8%) and previously being convicted of property crimes (23.9%).

**Table 6.** Comparison of covariates across classes.

Item	Class A	Class B	*P* value
Most probable class membership [*n* (%)]	2 268 (74.6)	771 (25.4)	
Demographics			
** **Year of birth [mean (SD)]	1977 (12)	1972 (12)	<0.0001
** **Male sex (%)	99.7	99.5	0.2870
** **Age at first registration [mean (SD)]	31.2 (11.6)	35.3 (11.3)	<0.0001
** **Low education (%)	35.4	47.9	<0.0001
Country of birth			
** **Swedish born with Swedish born parents (%)	37.8	49.4	
** **Swedish born with one parent born in Sweden (%)	5.1	7.3	
** **Swedish born with no parent born in Sweden (%)	5.5	6.9	
** **Born outside Sweden (%)	51.6	36.4	<0.0001
Socioeconomic status			
** **Neighbourhood deprivation [SDI (SD)]	1.21 (2.31)*	1.45 (2.40)*	<0.0001
** **Income [mean (SD)]	0.06 (1.04)*	−0.17 (0.86)*	<0.0001
** **Social welfare [*n* (%)]	26.0	51.9	<0.0001
Convicted crime			
** **Rape and aggravated rape (%)	79.8	92.7	<0.0001
** **Attempted rape and attempted aggravated rape (%)	20.6	7.9	<0.0001
Parents (*n* = 2 071)			
** **Convicted for the studied crimes (%)	0.5	0.5	0.948
** **Psychiatric disorder (%)	21.8	30.6	<0.0001
** **White collar crime (%)	14.3	20.3	0.0007
** **Property crime (%)	23.9	31.8	0.0002
** **Violent crime (%)	16.1	24.3	0.0007
** **Drug use disorder (%)	8.6	11.1	0.0739
** **Alcohol use disorder (%)	16.4	28.1	<0.0001
Resilience (*n* = 962) [mean (SD)]	−0.33 (0.97)*	−0.90 (1.00)*	<0.0001
IQ (*n* = 1 074) [mean (SD)]	−0.53 (0.97)*	−0.86 (0.92)*	<0.0001
School achievement (*n* = 1 093) [mean (SD)]	−0.82 (1.05)*	−1.52 (1.01)*	<0.0001

SD: standard deviation; SDI: social deprivation index. Note: one individual could be convicted of both crimes, thus the sum of the percentage is not 100%. *This is a standardized variable with mean 0 and SD 1. Please see more details in the Appendix 2.

Class B was also constituted of almost only men (99.5%). Those included in this class were somewhat older than those in Class A in regard to age at first registration as convicted, with a mean age of 35.3 (SD 11.3). Almost half of the offenders (47.9%) had a low education and the rate of immigrants (50.6%) in this class was somewhat lower than in Class A, although they still constituted the majority of the offenders. In line with Class A, the majority of the offenders had been convicted of rape and aggravated rape (92.7%), while the rest were convicted of attempted rape and attempted aggravated rape (7.9%). This class had a much higher proportion of offenders (in comparison to Class A) receiving social welfare (51.9%). Also, variables as resilience, IQ and school achievement were poorer than among those included in Class A. Close to one-third (30.6%) of the offenders had at least one parent with a psychiatric disorder and a substantial part of the offenders had at least one parent previously convicted of property crimes (31.8%) or violent crimes (24.3%).

### Validation of latent classes

Classes A and B were compared to each other in a multivariate logistic regression. The results showed that the risk for belonging to Class B was higher for individuals with a prior violent crime registration, an alcohol use disorder registration as well as individuals with low education, social welfare, low income and residing in a deprived area. All other included variables had broad 95%CIs and showed no clear pattern (Table 7).

**Table 7.** Results from a multivariate logistic regression.

Item	Odds ratio (95%CI) (Class A=0, Class B=1)
Demographics	
** **Year of birth	0.97 (0.96, 0.98)
** **Men *vs.* female	1.32 (0.31, 5.60)
** **Low education	1.80 (1.42, 2.72)
** **Age at first registration	1.02 (0.99, 1.05)
Country of birth	
** **Swedish born with Swedish born parents	Reference
** **Swedish born with one parent born in Sweden	1.36 (0.91, 2.02)
** **Swedish born with no parent born in Sweden	1.20 (0.78, 1.84)
** **Born outside Sweden	1.08 (0.79, 1.47)
Parents variables	
** **Convicted for the studied crimes	0.69 (0.15, 3.22)
** **Psychiatric disorder	0.95 (0.72, 1.26)
** **White collar crime	1.17 (0.84, 1.64)
** **Property crime	1.19 (0.89, 1.59)
** **Violent crime	1.52 (1.11, 2.08)
** **Drug use disorder	0.92 (0.60, 1.39)
** **Alcohol use disorder	1.68 (1.23, 2.30)
Neighbourhood deprivation	1.05 (1.00, 1.11)
Income	0.63 (0.55, 0.73)
Social welfare	3.27 (3.55, 4.19)

The two classes were further validated by evaluating their aggregation and co-aggregation within 8 101 363 Swedish full siblings (Table 8). The prevalence of sexual crimes in full siblings of the offenders in our two classes differed significantly (*P* < 0.0001) and was highest in siblings to offenders from Class A (0.52% ± 0.0%) and lowest in siblings to offenders from Class B (0.34% ± 0.0%). Table 8 presents the tetrachoric correlations among all full siblings in Sweden born 1950–1995. Evidence for a familial aggregation appeared in both Class A and B, with the former having slightly higher correlation (+0.25) than Class B (+0.23). Furthermore, the cross-class correlation was positive (+0.10), but with large standard error, suggesting some general familial aggregation of rape+. However, the overall pattern of correlations suggested some specificity in the familial factors influencing class membership.

**Table 8.** Tetrachoric correlations among all full siblings in the Swedish population born 1950–1995.

	Class A	Class B
Class A	0.25 (0.03)	0.10 (0.08)
Class B		0.23 (0.07)

### Additional sensitivity analysis

Some of the subjects in the LCA were not assigned to individual classes with high confidence. We repeated the analyses contained in Table 4 including only LCA assignments of greater probability than 70% (92% of the cases). Class membership declined more in Class A (10%), than in Class B (5%). Despite the reduction in numbers classified, the pattern of validators observed in Table 4 changed to a significant degree and the difference between classes remained, enhancing the overall robustness of our findings.

## Discussion

We aimed to study offenders convicted of rape+ against adult females in Sweden based on LCA. The study had two main findings: (1) Two classes appeared in the LCA where a majority of the rape+ offenders included in Class A were low offenders in comparison with those in Class B who were deemed to be high offenders; (2) A majority of the studied sample was constituted by first- and second-generation immigrants.

The majority of rape+ offenders (*n* = 2 268; 74.6%) in our cohort were found in Class A, which we deemed to be the “low offenders’ class”. Individuals in this class had in comparison with those in Class B (the high offenders’ class) substantially fewer rates of criminal convictions, psychiatric illness and substance use disorders.

Unfortunately, studies on rape offenders’ characteristics in a Swedish context are absent and, as far as we are aware, there is only one previous study, which has evaluated this issue in regard to adult male-on-female rape offenders. Stiernströmer et al. [14] studied 21 convicted male-on-female rapists in Malmö, Sweden, and found that the largest group of the offenders were between 30 and 40 years old (*n* = 8; 38%) and that a majority were immigrants (*n* = 15; 71%). Two-thirds (*n =* 13; 62%) were either employed or had some kind of practice/traineeship, while two (10%) were unemployed. Most of these offenders were thus between 30 and 40 years old, immigrants and employed. Although these figures are based on a small sample and no firm conclusions can be made, they resemble the characteristics in our Class A.

Recently, we performed a study on Swedish offenders being convicted or suspected of homicide, attempted homicide, preparation to commit homicide or conspiration to commit homicide (together called homicide+) [15]. An individual can in accordance with the Swedish Penal Code be prosecuted of conspiration if he or she together with another individual decides to commit a crime, convinces another individual to commit a crime or him- or herself takes charge to commit a crime. A total of 14 466 individuals committed for these crimes between 2000 and 2015 were analyzed using LCA. We identified three different latent classes; the first class, just like Class A in this current study, was constituted of homicide+ offenders with low rates of previous criminality, substance use disorders and psychiatric disorders. The second and third classes, just like our Class B in the present study, were constituted by moderate and high offenders, respectively. This striking resemblance show that not only in deadly violence [15] but also in rape, one class seems to be constituted by “traditional” criminals most likely well known by the police (i.e. Class B in the current study), while another class (i.e. Class A in the current study) is constituted by offenders that most likely have a more organized lifestyle and less criminal activity than Class B.

Our results with one class being deemed as “low offenders’” are supported by international studies. Fox and DeLisi [16] studied 3 857 juvenile sex offenders using LCA and could identify four classes of which the first class had low rates of criminal activity and psychopathologies, in comparison to the other classes which had significantly higher levels of repeated felony arrests. The existence of a “low offenders’ class” has also been shown in previous studies on youth sexual offenders by Vaughn et al. [17]. In a study on almost 18 000 youth sexual offenders, the authors used LCA and identified four different classes of which the first class was called “normative class” constituting of “low levels of substance use, delinquency, and violence” [17]. According to Vaughn et al., [17,18] there is also a class constituting close to 5% of the most severe delinquent youth sexual offenders. Although the rate of 5% is smaller than our rate for Class B (28.1%), and the above studies have been focussed on solely youth sexual offenders, the patterns of low offenders and high offenders are highly viable also in these studies.

More studies are needed in this aspect and not least, in regard to the question whether there are any differences between these two classes and rape typology (i.e. intimate partner rape, gang rape, stranger rape, etc.).

The finding of a low offending class both in the present study as well as in other studies on rape [16–18] and homicide+ [15,19] implies a new challenge in regard to both criminal policy, crime preventive interventions and measurements as well as risk assessments. There are many studies and reports on more “traditional” criminals. Low offending criminals, however, are a challenge, and their specific characteristics must be examined to a much higher degree in order to enhance preventive efforts.

The question of immigrant status is of importance in the field of criminology in both discussing offenders [20,21] as well as victims [22,23] of a crime. To differentiate between first- and second-generation immigrant status is also important in order to design educational programmes for immigrants on the Swedish law concerning sexual crimes. This could include the question on consent in sexual relationships and that women who are married can refuse their partners. However, results should be interpreted with caution and avoid statements on causality as such statements could be misinterpreted and misused, particularly if contextual factors, such as socioeconomic status, marital status, psychiatric disorders and lack of integration are not taken into account. Lack of integration often includes poor language skills and being unemployed. There may also be other factors associated with our findings that have not been explored. For example, possible bias against immigrants at the policing and prosecution level is a factor that cannot be ignored.

Of our total cohort (*n* = 3 039), the majority (*n* = 1 800; 59.2%) were immigrants of which almost half (*n =* 1 451; 47.7%) were born outside of Sweden. The findings in our study are in line with previous Swedish studies [9,14,24,25]. Our findings are also in line with international studies from, among others, Switzerland [26], as well as Sweden’s neighbouring countries Norway [27,28], Finland [28] and Denmark [29].

The Swedish National Council for Crime Prevention, i.e. Brå, has previously published two reports on immigrants and criminality. The first report was published in 1996 and studied the years 1985–1989 [24]. The second and most recent report was published in 2005 and studied the years between 1997 and 2001 [9]. Both reports showed that immigrants were overrepresented as suspects for a crime in the criminal statistics. A serious limitation in both these studies is that they have only studied individuals suspected of a crime, and therefore there is no information about how many that eventually were convicted or acquitted for a crime. In discussing rape, the first report showed that 38% of those being suspected for rape were born outside of Sweden [24]. The second report studied the rates of rape and attempted rape and showed that individuals born outside of Sweden had far higher rates (22%) of suspicions than Swedes (4%) [9].

It is difficult to compare our results with these two reports since we have focussed on individuals being convicted and not solely being suspected. Furthermore, we have studied rape+ while the above reports have focussed on rape and attempted rape. However, our finding that immigrants are overrepresented and constitute a majority in rape+, are in line with the findings in these two reports.

A recent study [14] on all convicted male-on-female rapists (*n* = 21) in Malmö, Sweden’s third largest city, between 2013 and 2018, showed that 15 (71%) of the convicted rapists were immigrants. This rate is a far higher rate of immigrants than in our study, which probably in part could be explained by the following four reasons: (1) the previous study only focussed on rape and aggravated rape, (2) the study sample was small, (3) only one single city with a high proportion of foreign-born inhabitants was studied and (4) only convictions during a limited timeframe (2013–2018) were studied.

## Conclusion

A total of 3 039 individuals in Sweden convicted of rape+ against a female were examined. By using LCA, two classes could be identified; one class was constituted of low offenders and one class was composed by high offenders. We could furthermore show that first- and second-generation immigrants constituted the majority of the rape+ offenders. Our results warrant further studies in regard to contextual factors in relation to those immigrants committing rape+ as well as the characteristics of low offending criminals in rape+. The results are of particular interest for the police authority, as well as prison and probation services. The results in regard to psychiatric ill-health may be used by the healthcare in order to customize specific treatments for rape+ offenders. Our findings are of particular importance for crime preventive efforts. Very little, however, is known about the association between rape+ and different contextual factors among immigrants in Sweden. More studies are needed in order to both understand the causes of this overrepresentation and majority status in rape+, and whether there are any differences among various demographic groups so that appropriate crime preventive measures can be taken. A better understanding of the contextual factors lying behind our findings may help future victims but also prevent young males from becoming rapists by identifying protective contextual factors that may help in the preventive work.

## Supplementary Material

Supplemental Material
