# Supplementary material for: Swedish rape offenders — a latent class analysis
Source: Forensic Sci Res. 2021 Feb 22;6(2):124–32. doi: 10.1080/20961790.2020.1868681 (PMC8330751; doi:10.1080/20961790.2020.1868681)
Supplement: Supplemental Material [file TFSR_A_1868681_SM3317.zip › Appendix 1 edited.docx]

**Appendix 1. Definition of sexual crimes**

Sexual crimes are defined in the Swedish Criminal Code (1962:700). Part 2, Chapter 6 of the code, sexual offences, defines sexual crimes as following:

Rape; gross rape; negligent rape; sexual assault; gross sexual assault; negligent sexual assault; rape of a child; gross rape of a child; sexual exploitation of a child; sexual assault of a child; gross sexual assault of a child; sexual intercourse with a descendant; sexual intercourse with a sibling; exploitation of a child for sexual posing; gross exploitation of a child for sexual posing; exploitation of a child through the purchase of a sexual act; sexual molestation; contact with a child for sexual purposes; purchase of sexual services; procuring; gross procuring; attempting to commit rape, gross rape, sexual assault, gross sexual assault, rape of a child, gross rape of a child, sexual exploitation of a child, sexual assault of a child, gross sexual assault of a child, exploitation of a child for sexual posing, gross exploitation of a child for sexual posing, exploitation of a child through the purchase of a sexual act, purchase of sexual services, procuring, gross procuring; preparation to commit procuring; preparation and conspiracy to commit, as well as to failure to disclose or prevent, rape, gross rape, rape of a child, gross rape of a child, gross exploitation of a child for sexual posing and gross procuring.
