# Supplementary material for: Swedish rape offenders — a latent class analysis
Source: Forensic Sci Res. 2021 Feb 22;6(2):124–32. doi: 10.1080/20961790.2020.1868681 (PMC8330751; doi:10.1080/20961790.2020.1868681)
Supplement: Supplemental Material [file TFSR_A_1868681_SM3317.zip › Appendix 2 edited.doc]

**Appendix 2: The definition of the variables used in this study**

| Variable | Definition |
| --- | --- |
| Psychiatric disorder | Any mental disorder; ICD10 codes: F00-F99, ICD8, 9: 290-319. Measured in the national inpatient register. Registrations for AUD and DA are not included.  Measured both in proband and in parent. For the **proband** the registration must occur prior to the Homicide registration. Treated as binary (yes/no) variable. For the **parents** it is lifetime registration. In the model treated as a binary (in any of the parent vs in none of the parents). |
| Drug abuse (DA) | DA was identified in the Swedish medical and mortality registries by ICD codes (ICD8: Drug dependence (304); ICD9: Drug psychoses (292) and Drug dependence (304); ICD10: Mental and behavioral disorders due to psychoactive substance use (F10-F19), except those due to alcohol (F10) or tobacco (F17)); in the Criminal Suspect Register by codes 3070, 5010, 5011, and 5012, that reflect crimes related to DA; and in the Crime Register by references to laws covering narcotics (law 1968:64, paragraph 1, point 6) and drug-related driving offences (law 1951:649, paragraph 4, subsection 2 and paragraph 4A, subsection 2). DA was identified in individuals (excluding those suffering from cancer) in the Prescribed Drug Register who had retrieved (in average) more than four defined daily doses a day for 12 months from either of Hypnotics and Sedatives (Anatomical Therapeutic Chemical (ATC) Classification System N05C and N05BA) or Opioids (ATC: N02A).  Measured both in proband and in parent. For the **proband** the registration must occur prior to the Homicide registration. Treated as binary (yes/no) variable. For the **parents** it is lifetime registration. In the model treated as a binary (in any of the parent vs in none of the parents). |
| Alcohol abuse (AUD) | AUD was identified in the Swedish medical and mortality registries by ICD codes: ICD9: V79B, 305A, 357F, 571A-D, 425F, 535D, 291, 303, 980; ICD 10: E244, G312, G621, G721, I426, K292, K70, K852, K860, O354, T51, F10); in the Crime Register by codes 3005, 3201, which reflect crimes related to alcohol abuse; in the Criminal Suspect Register by codes 0004, 0005 (Only those individuals with at least two alcohol-related crimes or suspicion of crimes from both Crime Register and Suspect Register were included); in the Prescribed Drug Register by the drugs disulfiram (Anatomical Therapeutic Chemical (ATC) Classification System N07BB01), acamprosate (N07BB03), and naltrexone (N07BB04).  Measured both in proband and in parent. For the **proband** the registration must occur prior to the Homicide registration. Treated as binary (yes/no) variable. For the **parents** it is lifetime registration. In the model treated as a binary (in any of the parent vs in none of the parents). |
| Violent crimial behaviour (VCB) | VCB was identified by registration in the Swedish Crime register and included (aggravated) assault, illegal threats, intimidation and illegal coercion, threats or violence against a police officer, (aggravated) robbery, kidnapping, arson, sexual crimes (excluding prostitution and the buying of sexual services but including child pornography).  Measured both in proband and in parent. For the **proband** the registration must occur prior to the Homicide registration. Treated as binary (yes/no) variable. For the **parents** it is lifetime registration. In the model treated as a binary (in any of the parent vs in none of the parents). |
| Property criminal behaviour (PCB) | PCB was identified by registration in the Swedish Crime register and included theft, vandalism, vandalism causing danger to the public, and trespassing).  Measured both in proband and in parent. For the **proband** the registration must occur prior to the Homicide registration. Treated as binary (yes/no) variable. For the **parents** it is lifetime registration. In the model treated as a binary (in any of the parent vs in none of the parents). |
| White collar criminal behaviour (WCB) | WCB was identified by registration in the Swedish Crime register and included fraud, forgery and dishonesty, and embezzlement.  Measured both in proband and in parent. For the **proband** the registration must occur prior to the Homicide registration. Treated as binary (yes/no) variable. For the **parents** it is lifetime registration. In the model treated as a binary (in any of the parent vs in none of the parents). |
| Low education | The highest achieved education in **proband**. Low education was defined as ≤9 years of schooling. |
| Income | Income is defined as the disposable income for the **proband** the year prior to the registration for homicide. In order to be able to compare the variable over time we have standardized the variable (mean 0 and std 1) by gender and year of birth. In the registers disposable income is defined as total personal income minus personal current taxes. |
| Social welfare | Social assistance is categorized into a binary variable based on whether or not the individual has received any social assistance during a year. Social assistance is defined as financial support under the Social Services Act. You can receive support for your upkeep and for other items that you need to have a reasonable standard of living. Examples of common situations when social assistance is given: As an income supplement to low-income families; for unemployed when other unemployment assistance is not provided or is insufficient; when sickness benefits are insufficient or not provided; to those who are bound by the children in the home and can not get childcare and therefore not can seek work. The variable is recorded at the family level, which means that all individuals in a family with social assistance will, in this report, be counted as recipients of Social assistance. The variable was measured the year prior to registration for homicide. |
| Neighborhood deprivation | All individuals reside in a SAMS area. There are approximately 9 200 SAMS throughout Sweden, with an average population of 1 000. These SAMS units were initially created by the Swedish authorities for administrative as well as marketing purposes. We created a neighborhood social deprivation index (SDI) for each of the SAMS neighborhoods based on register data for all residents in the neighborhood aged 25-64, i.e., the working-age population, which are assumed to have a stronger impact on the neighborhood than others. The SDI composite contained the following derived at baseline: the proportion of residents with low education (9 years or less), the proportion of residents with low household income (below half the median income), the proportion of unemployed residents, and the proportion of individuals on financial assistance. In the model the composite was kept as a continuous variable, with the SDI score ranging between -3 and 11 with higher values indicating greater levels of neighborhood deprivation. The variable was measured the year prior to registration for homicide. |
| Resilience | Measured at the Swedish military service conscription examination (at age 18-19). Resilience is designed to measure the ability to cope with psychologically stressful situations and is assessed on a 1 to 9 graded scale corresponding to a categorized normal distribution centered at 5. The score is assigned by a psychologist who conducts with every conscript a semi-structured conversation that averages 20–25 minutes. We standardized the variable per year with mean 0 and Std 1. Note that this variable was only measured in parts of the population. |
| School achievement | The National School Registry contained educational achievement (a grade point average) for all students at the end of grade nine (usually at age 16). From 1988 to 1997 the score was expressed on a scale between 1 (lowest) and 5 (overall mean was 3.2). From 1998 and onwards the score was expressed on scale between 10 (lowest) and 320 (overall mean was 207). For each year and by gender we standardized the grade score into a Z-score with mean 0 and SD 1. Note that this variable was only measured in parts of the population. |
| IQ | The Swedish military service conscription examination (at age 18-19) involves a full medical assessment including cognitive function (IQ) measured by four subtests representing logical, spatial, verbal and technical abilities. During the years covered by this study, this examination was required by law; only men of foreign citizenship or those with a severe medical condition or disability were excused. The global IQ score, derived from a summation of the four subtests, was standardized to give a Gaussian distributed score between one and nine. We translated this score into a standardized score with a mean of 1 and standard deviation of 0. |
| Country of birth | The variable was based on country of birth of both proband and parents and was categorized into 4 groups: Swedish born with Swedish born parents; Swedish born with one parent born in Sweden; Swedish born with no parent born in Sweden; Born outside Sweden. |
